# Association of physical activity with increased PI3K and Akt mRNA levels in adipose tissues of obese and non-obese adults

**DOI:** 10.1038/s41598-023-36365-0

**Published:** 2023-06-07

**Authors:** Marzieh Montazeri, Maryam Zarkesh, Azita Zadeh-Vakili, Davood Khalili, Monireh Movahedi, Alireza Khalaj

**Affiliations:** 1 Endocrine Research Center, Research Institute for Endocrine Sciences, Shahid Beheshti University of Medical Sciences, Tehran, Iran; 2 Cellular and Molecular Endocrine Research Center, Research Institute for Endocrine Sciences, Shahid Beheshti University of Medical Sciences, PO Box: 19395-4763, Tehran, Iran; 3 Endocrine Research Center, Research Institute for Endocrine Sciences, Shahid Beheshti University of Medical Sciences, PO Box: 19395-4763, Tehran, Iran; 4 Prevention of Metabolic Disorders Research Center, Research Institute for Endocrine Sciences, Shahid Beheshti University of Medical Sciences, Tehran, Iran; 5 Department of Biochemistry, Faculty of Biological Science, North Tehran Branch, Islamic Azad University, Tehran, Iran; 6 Tehran Obesity Treatment Center, Department of Surgery, Shahed University, Tehran, Iran

**Keywords:** Genetics, Molecular biology

## Abstract

Phosphatidylinositol-3-kinase (PI3K)/Akt signaling pathway regulates glucose and lipid metabolism. We examined the association of PI3K and Akt expression in visceral (VAT) and subcutaneous adipose tissue (SAT) with daily physical activity (PA) in non-diabetic obese and non-obese adults. In this cross-sectional study, we included 105 obese (BMI ≥ 30 kg/m^2^) and 71 non-obese (BMI < 30 kg/m^2^) subjects (aged/ ≥ 18 years). PA was measured using a valid and reliable International Physical Activity Questionnaire(IPAQ)-long-form, and the metabolic equivalent of task(MET) was calculated. Real-time PCR was performed to analyze the mRNA relative expression. VAT PI3K expression had a lower level in obese compared to non-obese (P = 0.015), while its expression was higher in active individuals than inactive ones (P = 0.029). SAT PI3K expression was increased in active individuals compared to inactive ones (P = 0.031). There was a rise in VAT Akt expression in the actives compared to the inactive participants (P = 0.037) and in non-obese/active compared to non-obese/inactive individuals (P = 0.026). Obese individuals had a decreased expression level of SAT Akt compared to non-obsesses (P = 0.005). VAT PI3K was directly and significantly associated with PA in obsesses (β = 1.457, P = 0.015). Positive association between PI3K and PA suggests beneficial effects of PA for obese individuals that can be partly described by PI3K/Akt pathway acceleration in adipose tissue.

## Introduction

Obesity is a global pandemic caused by excessive or abnormal accumulation of fat. The risk of diabetes mellitus, cardiovascular disease, hypertension, and hyperlipidemia is significantly increased in obese individuals^1^. The incidence of obesity has doubled in the last 30 years^2^, and researchers predict that 89% of men and 85% of women will be overweight or obese in 2030^3^. Owing to the growing prevalence of obesity, which is known as a socio-economic problem related to urbanization, it is necessary to find an effective way to prevent and treat it.

One of the significant ways to prevent obesity is making lifestyle changes, including increasing physical activity. Physical activity (PA) helps maintain energy balance by increasing energy consumption and may lead to weight loss if followed by a low-calorie diet^4^. Evidence suggests that PA and exercise training develop metabolic adaptations in multiple tissues, especially in the skeletal muscles and adipose tissue^5,6^. They also improve glucose homeostasis, which together has beneficial effects on the improving many metabolic and hormonal disorders besides the overweight and obesity^7^, including insulin resistance^8,9^, blood pressure^10^, and even stress-related disorders^11^. The phosphatidylinositol-3-kinase(PI3K)/Akt signaling pathway plays an essential role in glucose homeostasis and regulation of fat metabolism^12,13^; therefore, the relationship of this pathway with PA has been considered.

PI3K is a family of lipid kinases that phosphorylates phosphatidylinositol-3,4-diphosphate at the plasma membrane in response to activation by growth factors, insulin, cytokines, and hormones^14^. Insulin is the primary ligand for PI3K; its binding activates PI3K/Akt signaling pathway functions, resulting in increased glucose transporters mainly at the skeletal muscle cells and adipocytes^12^. Reduced expression of the alpha subunit of PI3K in rat adipose tissue, the most basic catalytic subunit of class 1A of this molecule, is associated with increased adipocytes and glucose intolerance^15^. Akt, protein kinase B, which regulates glucose and lipid metabolism, is divided into three isoforms based on serine and threonine amino acid differences^16^. Akt2 is mainly expressed in insulin-sensitive tissues, such as skeletal muscle, adipose tissue, and liver^17^. Akt protein plays a role in inhibiting the ectopic expansion of adipose tissue by inducing adipogenesis through the upregulation of PPARγ. Akt is also interplaying with Mammalian Target of Rapamycin (mTOR) and Sterol Regulatory Element-Binding Proteins (SREBPs) pathways^18,19^.

Considering the role of the PI3K/Akt signaling pathway in underlying mechanisms involved in lipid metabolism regulation, a comprehensive understanding of this pathway, its downstream molecules, and environmental factors affecting the regulation of involved genes expression can be beneficial in finding new ways to prevent and treating obesity.

Previous studies have shown alteration in PI3K/Akt pathway gene expression and protein levels following exercise in different tissues related to metabolic disorders. Nadi et al. showed that exercise increases the PI3K and Akt mRNA levels in nerve-muscle of diabetic rats^20^. Huang et al. and Li et al. detected increased levels of PI3K, Akt, and proteins after exercise training in the myocardial tissue of Wistar rats (compared to the sedentary group)^21,22^. Wang et al. showed that exercise increased the relative expression of PI3K and Akt proteins and activated PI3K/Akt signaling pathway in (hippocampus of) rats' brain^23^. Despite the critical effect of PA on adipose tissue (metabolic adaptation and changes in size and fat content), few studies on the association between PA and PI3K and Akt gene expression in adipose tissue^15,24^. Therefore, in the present study, we investigated the relationship between PI3K and Akt expression with daily PA in adipose tissues of non-diabetic obese and non-obese adults. VAT and SAT were examined due to reported differences in their metabolic effect^6^ and their response to PA^25^.

## Materials and methods

### Participants

In this cross-sectional study, 176 individuals (134 women and 42 men) aged 19–69 years were selected among patients who were admitted to the Mostafa Khomeini and Khatam Al-Anbia hospitals, (Tehran, Iran) for minimal abdominal surgery including appendectomy and hernia repair for both obese and non-obese patients between 2012 and 2015. People with diabetes or cancer and women who were pregnant or breastfeeding were excluded. Demographic information, history of medicine were collected. Before surgery, 5 ml of fasting blood samples were gathered for biochemistry measurements. Subcutaneous and visceral adipose tissues (SAT and VAT) were collected by a single expert surgeon during elective surgery. During the surgery, SAT was harvested from the 5 cm periumbilical area; VAT was collected from the upper left part of omentum (subomental) fat, directly frozen in liquid nitrogen, and stored at − 80 °C. This study was conducted following the Declaration of Helsinki and RIES institutional guidelines. Written informed consent was obtained from all the participants.

This research was approved by the Ethics Committee of the Research Institute for Endocrine Sciences (RIES) of Shahid Beheshti University of Medical Sciences (No. IR.SBMU.ENDOCRINE.REC.1399.083).

### Anthropometric and laboratory measurements

Weight, height, waist, hip, and neck circumference were measured according to standard protocols described in detail previously^26^. Body mass index (BMI) was calculated as weight (kg) divided by the square of height (m^2^), and participants were divided according to their BMI status into two groups, including obese with BMI ≥ 30 kg/m^2^ and non-obese with BMI < 30 kg/m^2^. After 15 min of rest, systolic and diastolic blood pressures (SBP and DBP) were measured twice in one-minute intervals with a mercury sphygmomanometer, and their mean was recorded^27^.

All serums were gathered after centrifuging the fasting blood samples at 3000×*g* for 5 mins. An enzymatic colorimetric method with glucose oxidase was used to measure fasting plasma glucose (FPG), and the inter-and intra-assay coefficients of variation (CV) were both 1.0%. Triglyceride (TG) levels were measured by enzymatic calorimetry using glycerol phosphate oxidase, and there were 0.4 and 2.1% CVs between inter-and intra-assays of TG. Total cholesterol (TC) was assayed with the cholesterol esterase and cholesterol oxidize method; inter-and intra-assay CV of TC were 0.5 and 1.7%, respectively. The measurements of FPG, TG, and TC levels were performed using commercial kits (Pars Azmoon Inc., Tehran, Iran). The enzyme-linked immunosorbent assay (ELISA) measured insulin levels (Mercodia kits, Uppsala, Sweden). The inter-and intra-assay CVs of insulin were 1.7 and 2.3%.

### Assessment of physical activity

The validated International Physical Activity Questionnaire (IPAQ)-long-form^28^ was used to accumulate participants' PA during seven days whose reliability, validity, and reproducibility have been assessed in Iranian society^29^. The IPAQ data was converted into metabolic equivalents (MET-min/1 week) for each type of activity by multiplying the number of minutes allocated to each activity by the associated MET score (One MET is equal to energy expenditure during rest and is approximately equal to 3.5 ml O2/kg/min in adults). Therefore, participants were categorized into active (≥ 600 MET-minutes/week) and inactive (< 600 MET-minutes/week) groups^30^.

### Evaluation of genes expression in adipose lesions

Tissue samples were weighted and incised before RNA extraction. Totally, 30–50 mg visceral and subcutaneous fat tissues were added to 1 mL TRIzol reagent (Invitrogen, U.S.). Total RNA was extracted based on the manufacturer’s instructions. Proteins, lipids, carbohydrates, and cell debris were eliminated through the extraction of the aqueous. Then, the Nanodrop spectrophotometer (ND-1000, Thermo Scientific, USA) was used to measure the 260/280 and 260/230 nm absorption ratios to evaluate the quality of the extracted RNA. cDNA was synthesized from total RNA according to the instruction of the cDNA synthesis kit (BIOFACT, South Korea). GAPDH (housekeeping gene) was used as an internal control gene in a real-time quantitative PCR instrument (qPCR) Rotor-Gene 6000 (Corbett Life Sciences, Sydney, Australia).

The primer sequences of PI3K, Akt and GAPDH were as follows: PI3K (NM_006218.4) Forward: 5′-CAG AAC AAT GCC TCC ACG A-3′, Reverse: 3′-CAC GGA GGC ATT CTA AAG TC-5′ with product size 122 bp; Akt (NM_001382431.1) Forward: 5′-CAC TTT CGG CAA GGT GAT CC-3′, Reverse: 3′-GTC CTT GGC CAC GAT GAC TT-5′ with product size 94 bp, and GAPDH (NM_001357943.2) Forward: 5′-CTG CTC CTC CTG TTC GAC AGT-3′, Reverse: 3′-CCG TTG ACT CCG ACC TTC AC-5′ with product size 100 pb. The annealing temperature for the PI3K and Akt was determined to be 60 and 61 °C, respectively.

The qPCR was performed in 20 μL volumes containing 1 μL of the cDNA, 1 μL forward primers, 1 μL reverse primers, 10 μL 2X SYBR Green Master mix (BIOFACT, South Korea), and 7 μL DEPC water. Then samples were run in duplicate for target genes and GAPDH with the thermal cycling conditions: 10 min at 95 °C, followed by 40 cycles 30 s at 95 °C, 30 s at 60 °C (for PI3K) or 30 s at 61 °C (for Akt) and 30 s at 72 °C. The reference gene normalized the mRNA levels of PI3K and Akt in each sample. The relative quantitation expression was performed by the comparative Ct method according to Livak et al.^31^ according to the following formula:

$$\Delta {\text{Ct}}_{{{\text{obese}}}} = {\text{ Ct}}_{{({\text{PI3K }}\;{\text{or }}\;{\text{Akt}})}} - {\text{ Ct}}_{{({\text{GAPDH}})}}$$

$$\Delta {\text{Ct}}_{{\text{non - obese}}} = {\text{ Ct}}_{{({\text{PI3K}}\;{\text{ or }}\;{\text{Akt}})}} - {\text{ Ct}}_{{({\text{GAPDH}})}}$$

$$\Delta \Delta {\text{Ct}} = \, \Delta {\text{Ct}}_{{{\text{obese}}}} - \, \Delta {\text{Ct}}_{{\text{non - obese}}}$$

$${\text{Relative}}\;{\text{expression}} = { 2}^{{ - \Delta \Delta {\text{Ct}}}}$$

### Statistical analysis

Statistical analyses were performed using SPSS (version 20) software. The Kolmogorov–Smirnov test was used to evaluate the normal distribution of variables. Normal variables including age, BMI, waist, wrist, hip, and neck circumference, fasting blood glucose, total cholesterol, and PA expressed as mean ± standard deviation (SD), and non-normal variables including SBP, DBP, and TG levels as median (interquartile 25, 75). Insulin was log-transformed to better approximate a normal distribution.

To compare quantitative variables between non-obese and obese groups, Independent-Sample T-test was used for variables with normal distribution, and the Mann–Whitney *U* test for variables with non-normal distribution. Analysis of variance (ANOVA) and tukey HSD post-hoc tests were carried out to compare the insulin levels between four studied groups. The Chi-Square test was also used to compare qualitative variables such as gender between non-obese and obese groups. Mann–Whitney *U* test was used to compare the expression of genes in two different tissues and between non-obese and obese groups. Comparative graphs of relative expression between study groups were drawn with GraphPad Prism8 software with Mean ± 1SEM.

Correlation between PI3K and Akt gene expression in VAT and SAT with PA was examined using the Pearson Correlation test. STATA software was used to confirm a linear relationship between gene expression and PA by the Restricted Cubic Splines model using the multivariate regression splines (mvrs) command.

The interaction of PA with BMI and sex on gene expression was tested through linear regression analysis. Due to the significant interaction between PA and BMI, the analysis was performed for two distinct groups (physically active and inactive). Furtheremore, there was no significant interaction between PA and sex in gene expression. The association of PA and gene expression was also examined using linear regression analysis with age and sex adjustments.

## Results

### Characteristics of the participants

This study evaluated 176 adults (aged 19–65 years). Seventy-one subjects (50 women, 21 men) who had a mean age of 48.3 ± 14.9 years were in the non-obese group, and 105 subjects (84 women, 21 men) who had a mean age of 37.0 ± 10.7 years were in the obese group. Information on anthropometric, biochemical, and PA variables in non-obese and obese individuals is shown in Table 1. Non-obese individuals had a higher mean age than the obese group. The mean BMI, waist, wrist, hip and neck circumference, FBS, total cholesterol, and median of the SBP, DBP, TG, and insulin levels were also higher in obese subjects than in non-obese participants. Although the mean MET of non-obese individuals was higher compared to obese ones, this was not statistically significant (Table 1). Results showed no differences in insulin levels between active and inactive individuals neither in obese nor non-obese groups based on the ANOVA Post-hoc (Tukey HSD) test. The significant ANOVA P-value is due to the difference in insulin levels between obese and non-obese subjects (Table 2).

**Table 1:** Anthropometric, biochemical, and PA characteristics in non-obese, obese, and total individuals.

	Non-obese (BMI < 30 kg/m^2^) (n = 71)	Obese (BMI ≥ 30 kg/m^2^) (n = 105)	Total (n = 176)	P value
Age (years)	48.3 ± 14.9	37.0 ± 10.7	41.4 ± 13.6	< 0.001
Sex				
Male	21 (29.6%)	21 (20.0%)	42 (23.9%)	0.154
Female	50 (70.4%)	84 (80.0%)	134 (71.6%)	
Body mass index (kg/m^2^)	24.6 ± 2.8	43.1 ± 6.1	35.6 ± 10.4	< 0.001
Waist Circumference (cm)	87.2 ± 12.9	121.7 ± 16.5	107.8 ± 22.7	< 0.001
Wrist circumference (cm)	17.3 ± 1.4	19.2 ± 2.1	18.6 ± 2.1	< 0.001
Hip circumference (cm)	97.5 ± 13.9	132.1 ± 15.9	118.2 ± 22.8	< 0.001
Neck circumference (cm)	35.7 ± 3.7	41.2 ± 4.3	39.4 ± 4.8	< 0.001
Systolic blood pressure (mmHg)	110.0 (107.5–120)	120.0 (110–120)	120.0 (110–120)	0.018
Diastolic blood pressure (mmHg)	70.0 (70.0–80.0)	80.0 (70.0–80.0)	77.5 (70.0–80.0)	0.032
Fasting plasma glucose (mg/dl)	84.8 ± 20.0	91.3 ± 18.5	88.7 ± 19.3	0.027
Cholesterol (mg/dl)	167.1 ± 44.2	184.5 ± 37.4	177.4 ± 41.1	0.005
Triglycerides (mg/dl)	79.0 (64.0–127.9)	109 (71.5–155.0)	101 (67.2–148.7)	0.002
Insulin (mg/dl)	5.1 (2.7–10.6)	12.5 (6.1–22.9)	8.6 (4.4–19.5)	< 0.001
Physical activity (MET-min/wk)	2327.2 ± 514.4	1481.3 ± 286.5	1822.5 ± 269.8	0.154

Qualitative data are reported as percent; Normal and non-normal variables were expressed as mean ± standard deviation (SD) and median (IQ 25–75), respectively. Independent-Sample T-test and Mann–Whitney U test were used to compare quantitative normal and non-normal variables, respectively. The Chi-Square test was used to compare qualitative variables between the two groups.

**Table 2:** Comparison of the insulin levels between four studied groups.

	Insulin (mg/dl)	P_(Tukey)_	P value
Non-obese			
Inactive	5.81 ± 2.63	0.976	< 0.001
Active	5.35 ± 2.48		
Obese			
Inactive	11.80 ± 2.23	0.975	
Active	11.00 ± 2.15		

Analysis of variance (ANOVA) and tukey HSD post-hoc tests were carried out to compare the insulin levels between four studied groups.

### PI3K gene expression analysis

**Figure 1:**
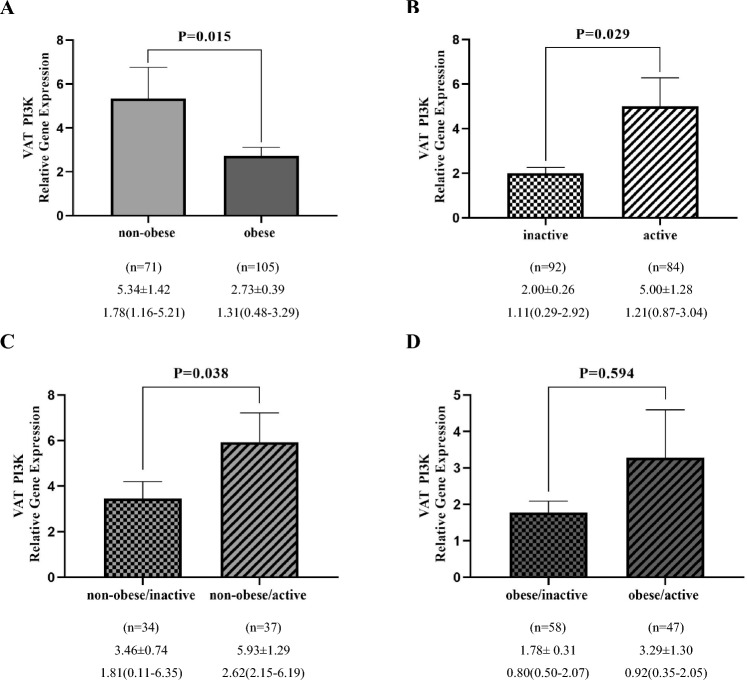
Comparison of relative expression of PI3K gene in VAT between non-obese and obese groups (**A**), inactive and active (**B**), non-obese/inactive and non-obese/active (**C**), obese/inactive and obese/active (**D**). The numbers below the figures indicated the mean ± SEM and also median (Interquartile ranges 25–75%). The displayed P values obtained from the Mann–Whitney *U* test, which was used to compare the differences between two groups.

**Figure 2:**
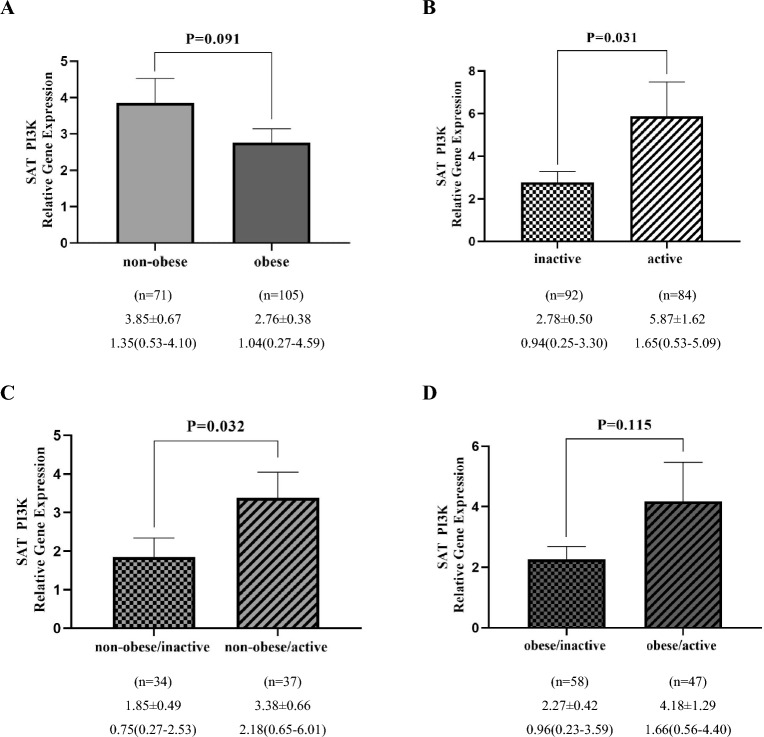
Comparison of relative expression of PI3K gene in SAT between non-obese and obese groups (**A**), inactive and active (**B**), non-obese/inactive and non-obese/active (**C**), obese/inactive and obese/active (**D**). The numbers below the figures indicated the mean ± SEM and also median (Interquartile ranges 25–75%). The displayed P values obtained from the Mann–Whitney *U* test, which was used to compare the differences between two groups.

Based on Mann–Whitney *U* test results, there was a significant decrease in the VAT PI3K gene expression in obese individuals compared to non-obese individuals (P = 0.015) (Fig. 1A). Moreover, it was upregulated significantly in active individuals compared to inactive ones (P = 0.029) and in non-obese/active subjects compared to non-obese/inactive individuals (P = 0.038) (Fig. 1B,C). However, VAT PI3K expression had no significant difference between obese/active and obese/inactive individuals (P = 0.594) (Fig. 1D). Moreover, The SAT PI3K mRNA level was not different between obese and non-obese individuals (P = 0.091) (Fig. 2A). Nevertheless, it was increased significantly in active individuals compared to inactive one (P = 0.031) and in non-obese/active participants compared to non-obese/inactive group (P = 0.032) (Fig. 2B,C). On the other hand, no significant difference was observed between obese/active and obese/inactive groups (P = 0.115) (Fig. 2D).

### Akt gene expression analysis

**Figure 3:**
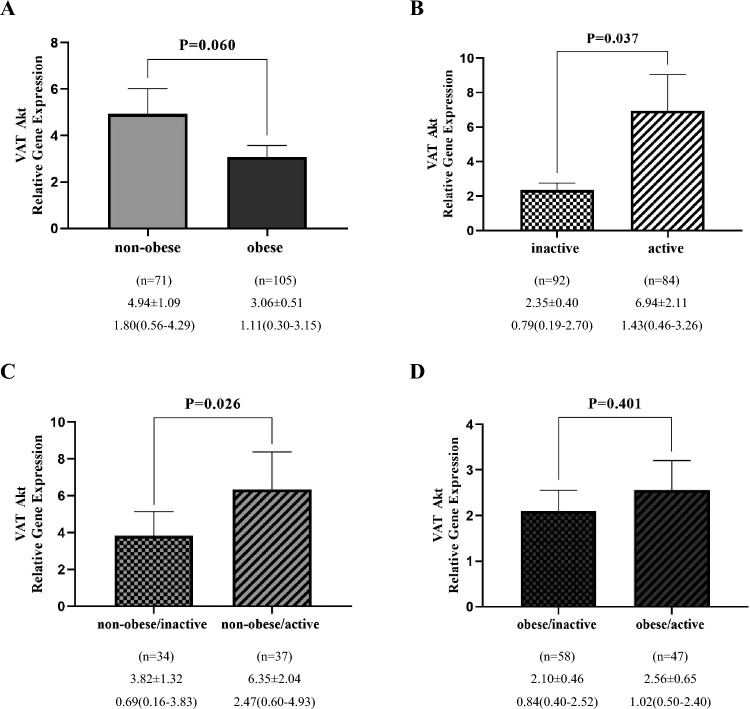
Comparison of relative expression of Akt gene in VAT between non-obese and obese groups (**A**), inactive and active (**B**), non-obese/inactive and non-obese/active (**C**), obese/inactive and obese/active (**D**). The numbers below the figures indicated the mean ± SEM and also median (Interquartile ranges 25–75%). The displayed P values obtained from the Mann–Whitney *U* test, which was used to compare the differences between two groups.

**Figure 4:**
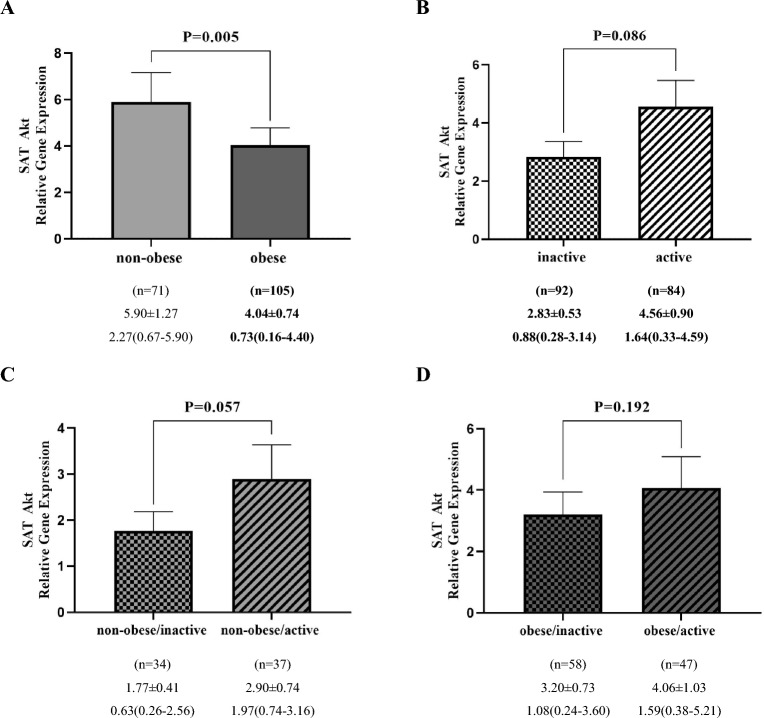
Comparison of relative expression of Akt gene in SAT between non-obese and obese groups (**A**), inactive and active (**B**), non-obese/inactive and non-obese/active (**C**), obese/inactive and obese/active (**D**). The numbers below the figures indicated the mean ± SEM and also median (Interquartile ranges 25–75%). The displayed P values obtained from the Mann–Whitney *U* test, which was used to compare the differences between two groups.

Gene expression levels were compared between groups using the Mann–Whitney *U* test. The results showed that VAT Akt mRNA level was marginally different between obese and non-obese groups (P = 0.060) (Fig. 3A). However, it was significantly raised in active group compared to inactive group (P = 0.037) and in non-obese/active compared to non-obese/inactive individuals (P = 0.026) (Fig. 3B,C). The expression of this gene was not significant between the obese/active group and obese/inactive subjects (P = 0.401) (Fig. 3D). Besides, SAT Akt expression was significantly decreased in obese individuals compared to non-obese one (P = 0.005) (Fig. 4A). It was elevated in active compared to inactive group (P = 0.086), in non-obese/active participants compared to non-obese/inactive one (P = 0.057) and in obese/active individuals compared to obese/inactive group (P = 0.192), which were not statistically significant (Fig. 4B–D).

### Correlation and linear regression analysis

The results of the Pearson Correlation test showed a positive correlation between PI3K and Akt expressions with PA in VAT of obese individuals (r = 0.254, P = 0.009 and r = 0.265, P = 0.006, respectively). However, no significant correlation was found between PI3K and Akt expressions with PA in VAT and SAT for non-obese group (Table 3).

**Table 3:** Correlation between PI3K and Akt expression and physical activity in VAT and SAT in non-obese, obese and total individuals.

	Non-obese (n = 71)		Obese (n = 105)		Total (n = 176)	
	r*	P value	*r*	P value	*r*	P value
Visceral adipose tissue						
PI3K	− 0.094	0.437	0.254	0.009	0.078	0.301
Akt	− 0.048	0.688	0.265	0.006	0.111	0.141
Subcutaneous adipose tissue						
PI3K	0.035	0.769	0.107	0.278	0.073	0.337
Akt	0.061	0.615	0.015	0.879	0.039	0.607

The linear relationship between the variables was measured using Pearson Correlation test. r*: correlation coefficient.

VAT PI3K expression is directly and significantly associated with PA in obese individuals (β = 1.457, P = 0.015), using linear regression analysis. However, other groups had no significant association between PA with VAT and SAT PI3K and Akt levels. Besides, there was no significant effect of sex and age (Table 4).

**Table 4:** The association between PI3K and Akt gene expression and physical activity in VAT and SAT of non-obese and obese groups.

	VAT		SAT	
	PI3K β (95% CI)	Akt β (95% CI)	PI3K β (95% CI)	Akt β (95% CI)
Non-obese				
PA	− 1.40 (− 3.12, 0.33)	− 1.07 (− 2.82, 0.69)	1.02 (− 0.88, 2.92)	1.11 (− 0.93, 3.16)
Age	− 0.02 (− 0.08, 0.04)	− 0.01 (− 0.07, 0.05)	− 0.01 (− 0.07, 0.06)	− 0.01 (− 0.08, 0.06)
Sex	1.61 (− 0.26, 3.48)	1.88 (− 0.02, 3.77)	0.31 (− 1.74, 2.37)	0.02 (− 2.20, 2.23)
Obese				
PA	1.46 (0.29, 2.62)*	1.06 (− 0.20, 2.33)	0.62 (− 0.81, 2.05)	0.60 (− 0.72, 1.92)
Age	− 0.05 (− 0.10, 0.00)	− 0.05 (− 0.11, 0.00)	0.00 (− 0.06, 0.07)	0.01 (− 0.06, 0.07)
Sex	0.46 (− 0.98, 1.91)	0.37 (− 1.21, 1.94)	0.29 (− 1.49, 2.06)	0.25 (− 1.89, 1.39)

The P values obtained from linear regression analysis after sex and age adjustment. *PA* physical activity, *VAT* visceral adipose tissue, *SAT* subcutaneous adipose tissue, *CI* confidence interval, *P value < 0.001.

## Discussion

The present study showed that VAT PI3K expression was different between obese and non-obese participants according to their activity and inactivity. This gene's relative expression was lower in the obese group than the non-obese group (regardless of activity status). However, it was higher in active subjects than inactive ones (regardless of BMI status). In non-obese individuals, daily activity (MET) was associated with a higher expression of PI3K. In obese individuals, PA showed a significant positive correlation with PI3K expression. Increased SAT PI3K expression level was also observed in the active group compared to the inactive group and in active obese subjects compared to the inactive obese ones.

Regarding the Akt gene, the expression changes of this gene were observed in both VAT and SAT. Higher Akt mRNA level was shown in active individuals compared to inactive subjects (regardless of BMI status) and the non-obese/active subjects compared to non-obese/inactive counterparts in VAT. Moreover, in the obese group, PA had a significant positive correlation with Akt expression. SAT Akt expression was significantly decreased in obese people compared to non-obese people (regardless of activity status).

In 2005, Peres et al. studied the relationship between endurance exercise training and adipocytes' response to insulin in Wistar rats^24^. Male rats ran on a treadmill for one hour a day for seven weeks compared to age-matched sedentary controls. At the end of the study, the final weight of rats in the two groups was not significantly different. The epididymal adipocytes were smaller in the trained group, and plasma glucose and insulin levels were lower than in the control group. Insulin-induced glucose uptake, PI3K-dependent phosphorylation in insulin receptors-1 and 2, and Akt (Ser 473) were also higher in the training group than in the control group. For the first time, the researchers attributed the improved adipocyte insulin responsiveness following exercise to the modulatory effect of the PI3K/Akt signaling pathway.

Our results in this study show a significant decrease in PI3K expression in inactive individuals compared to active ones, regardless of their obesity status. During PA, glucose uptake increases in adipose tissue, and therefore, it is necessary to increase the glucose transporter 4 (GLUT4) at the adipocyte surface^32,33^. GLUT4 is the insulin-responding glucose transporter, a major mediator of glucose removal from the circulation, and a key regulator of whole-body glucose homeostasis. The mechanism of this increase in adipose tissue is unknown, but the consequence of PI3K/Akt pathway activation via insulin is the rise of GLUT4 transportation to the cell surface. Therefore, it seems logical that PA is associated with a higher level of genes expressed in this pathway. However, Ferrannini et al. indicated that insulin-dependent glucose uptake in VAT following exercise was not different between insulin-resistant and non-insulin-resistant individuals. Therefore, according to this result, they attributed the increased glucose uptake in this tissue to the non-insulin-dependent mechanisms^34^.

On the other hand, a study in 2019 showed that the treatment of cultured human adipocytes (isolated from subcutaneous abdominal adipose tissue) with obtained serum from subjects after 60 min of cycling exercise elevated the expression of GLUT4. They suggest circulating factors mediate exercise-induced effects on adipose tissue GLUT4 expression^35^. The origin and identity of this serum transmitted potential factors are still unknown. Since PI3K can be activated by non-insulin ligands, including hormones and growth factors^36^, a rise in their serum concentration occurs after exercise, PI3K may play a role in the initiation of the pathway and increase glucose uptake.

One of the important topics investigated in this study was the evaluation of PA effect on gene expression in both SAT and VAT. In addition to anatomical position and mechanical behavior; SAT and VAT have metabolic differences. The adipocytes in the VAT are metabolically more active, more sensitive to lipolysis and more insulin-resistant than SAT adipocytes. VAT is also associated with overstated inflammatory, which explains close association between VAT accumulation and cardiometabolic risk and morbidity^37,38^. Moreover, PI3K/Akt axis expression pattern was not studied in human VAT and SAT separately. We assumed that the response of PI3K/Akt genes expression in VAT might be different from their expression in SAT. Therefore, we collected both adipose tissues. In this study, based on the relative gene expression analysis, the results indicated that PI3K mRNA levels in VAT and the Akt mRNA levels in SAT of obese subjects significantly decreased. Meanwhile, PI3K expression increased in both SAT and VAT of subjects with higher PA. This may indicate that PA has the same effect on PI3K mRNA level in both fat tissues. On the other hand, the relationship between PA and Akt expression was observed only in VAT, which can indicate the greater effect of PA on the Akt expression inVAT. The present study revealed that the difference in the expression levels of the studied genes was more prominent in VAT than SAT. Likely, intrinsic depot-specific differences such as higher androgen receptor levels, lower leptin secretion, and mRNA expression, greater glucocorticoid density, and greater IL-6 secretion may play a role in how PA affects them^39^. Quantifying fatty acid and glucose uptake using positron emission tomography (PET) has shown that VAT is metabolically more active than SAT^40,41^. Currently, it is hypothesized that the dysfunction of VAT plays a significant role in obesity pathophysiology; due to its higher level of adrenergic activity, it responds more to PA changes^30–32^. The effect of PA on VAT reduction can be partly explained by the IL-6, which is secreted from the contracting muscle and mainly stimulates lipolysis in VAT^42,43^. IL-6 functions as an anti-inflammatory cytokine and is one of the first cytokines to be upregulated after exercise^44,45^. It has been shown that IL-6 receptor blockade impairs this exercise-induced lipolysis^46^.

In 2020, Meng et al. showed that extracted flavonoids from mulberry leaf extract improved hyperglycemia, glycolipid metabolism, adiponectin, and leptin secretion in the insulin-resistant model of 3T3-L1 adipocytes^47^. They revealed this metabolic improvement was associated with increased expression of PI3K, Akt, and IRS1 proteins and thus activation of the PIK3/Akt pathway and high membrane transfer capacity of GLUT4. Wartmannin, a PI3K inhibitor, significantly reduced glucose uptake and secretion of leptin and adiponectin. Therefore, increased expression of PIK3/Akt genes was associated with better glycolipid metabolism, glucose uptake, and adiponectin secretion.

In concordance with these findings, we recognized lower Akt expression in SAT of obese participants than non-obese and VAT of inactive individuals (obese and non-obese) compared to active ones. There was also a positive correlation between the expression of the Akt gene and PA in obese participants. Findings may verify the PIK3/Akt pathway mediatory role in responding to PA.

Adiponectin is one of the essential hormones in adipose tissue, and the reduction of its expression is directly related to the incidence of obesity^48^. This adipokine is recognized as a potential biomarker for the beneficial effects of PA^49^. Pereira et al. observed a decrease in insulin-stimulated adiponectin secretion in 3T3-L1 adipocytes following inhibition of PI3K using a kinase-inactive Akt adenoviral construct or treatment with Wortmannin^50^. A positive correlation between PI3K expression and PA in our study suggests that perhaps part of the health benefits of PA for obese individuals is due to an increase in adiponectin secretion via PI3K/Akt pathway acceleration.

Based on previous studies and as we expected, increased adipose tissue suppresses the PI3K/Akt signaling pathway. The genes of this pathway are less expressed in obese people than in non-obese individuals. Although mRNA levels alone are not always sufficient to predict protein levels, as a critical indicator of the regulation of gene expression, it shows the development of an adaptive response. In a recent study on children with different degrees of obesity, the levels of PI3K mRNA and Akt mRNA in peripheral blood mononuclear cells were lower in each obesity group than in the control group^51^. In another study on the blood samples of diabetic patients, PI3K and Akt2 mRNA levels were significantly lower in the obese diabetic group compared to other groups^52^.

Insulin is the main activator of PI3K-Akt signaling pathway. Binding to its transmembrane receptor, insulin promotes the signaling cascade, leading to GLUT4 translocation to the cellular membrane and glucose uptake^53^. This process explains lower PI3K and Akt expression in the obese group with higher insulin and FBS levels in serum.

On the other hand, considering the importance of this pathway in increasing glucose uptake and PA's role in energy consumption, it seems that PA has a role in improving obesity status by affecting the genes of the PI3K/Akt pathway. The association between PA and VAT PI3K gene expression in the obese group has been shown better after adjustment for age and sex. This observation revealed the importance of PA.

According to the literature, this study is the first study investigating the relationship between PA and PI3K/Akt gene expression in VAT and SAT of human samples. The limitations of this study include: due to limited funding, it was not possible to measure these genes' protein production. It was not possible to examine this pathway's upstream and downstream genes. Collecting more tissue specimens for matching was impossible due to the study on human samples.

The results of this cross-sectional study can be used to design experimental studies; in the clinical trial, studies to investigate the effect of PA on gene expression of this pathway in fatty tissues.

## Data Availability

The datasets used and analyzed in the present study are available from the corresponding author on reasonable request.

## References

[1] Panuganti K, Nguyen M, Kshirsagar R (2021). Obesity.

[2] Baxter J (2019). Updates on monogenic obesity in a multifactorial disease. Obes. Surg..

[3] Engin A (2017). The definition and prevalence of obesity and metabolic syndrome. Adv. Exp. Med. Biol..

[4] Wareham N (2007). Physical activity and obesity prevention. Obes. Rev..

[5] Thyfault JP, Bergouignan A (2020). Exercise and metabolic health: Beyond skeletal muscle. Diabetologia.

[6] Kolnes KJ, Petersen MH, Lien-Iversen T, Højlund K, Jensen J (2021). Effect of exercise training on fat loss-energetic perspectives and the role of improved adipose tissue function and body fat distribution. Front. Physiol..

[7] Oppert JM, Bellicha A (2021). Exercise training in the management of overweight and obesity in adults: Synthesis of the evidence and recommendations from the European Association for the Study of Obesity Physical Activity Working Group. Obesity.

[8] Sampath Kumar A (2019). Exercise and insulin resistance in type 2 diabetes mellitus: A systematic review and meta-analysis. Ann. Phys. Rehabil. Med..

[9] Iaccarino G (2021). Modulation of insulin sensitivity by exercise training: Implications for cardiovascular prevention. J. Cardiovasc. Transl. Res..

[10] Bersaoui M, Baldew SM, Cornelis N, Toelsie J, Cornelissen VA (2020). The effect of exercise training on blood pressure in African and Asian populations: A systematic review and meta-analysis of randomized controlled trials. Eur. J. Prev. Cardiol..

[11] Lee HJ, Baek SS (2017). Role of exercise on molecular mechanisms in the regulation of antidepressant effects. J. Exerc. Rehabil..

[12] Huang X, Liu G, Guo J, Su Z (2018). The PI3K/AKT pathway in obesity and type 2 diabetes. Int. J. Biol. Sci..

[13] Yang H (2020). Fluoxetine regulates glucose and lipid metabolism via the PI3K-AKT signaling pathway in diabetic rats. Mol. Med. Rep..

[14] Burke JE, Williams RL (2015). Synergy in activating class I PI3Ks. Trends Biochem. Sci..

[15] Nelson VL, Jiang Y-P, Dickman KG, Ballou LM, Lin RZ (2014). Adipose tissue insulin resistance due to loss of PI3K p110α leads to decreased energy expenditure and obesity. Am. J. Physiol. Endocrinol. Metab..

[16] Abeyrathna P, Su Y (2015). The critical role of Akt in cardiovascular function. Vascul. Pharmacol..

[17] Krycer JR, Sharpe LJ, Luu W, Brown AJ (2010). The Akt-SREBP nexus: Cell signaling meets lipid metabolism. Trends Endocrinol. Metab..

[18] Cai H, Dong LQ, Liu F (2016). Recent advances in adipose mTOR signaling and function: Therapeutic prospects. Trends Pharmacol. Sci..

[19] Lee PL, Tang Y, Li H, Guertin DA (2016). Raptor/mTORC1 loss in adipocytes causes progressive lipodystrophy and fatty liver disease. Mol. Metab..

[20] Nadi M, Banaeifar A, Arshadi S (2021). Effect of an aerobic exercise course on PI3K and AKT1 expression and neural muscle insulin resistance in diabetic rats. Iran. J. Diabetes Obes..

[21] Huang CY (2012). Anti-apoptotic and pro-survival effects of exercise training on hypertensive hearts. J. Appl. Physiol..

[22] Li J, Xu P (2020). Exercise preconditioning plays a protective role in exhaustive rats by activating the PI3K-Akt signaling pathway. Evid. Based Complement. Altern. Med..

[23] Wang LR, Baek SS (2018). Treadmill exercise activates PI3K/Akt signaling pathway leading to GSK-3β inhibition in the social isolated rat pups. J. Exerc. Rehabil..

[24] Peres SB (2005). Endurance exercise training increases insulin responsiveness in isolated adipocytes through IRS/PI3-kinase/Akt pathway. J. Appl. Physiol..

[25] Abe T (2022). Comparisons of calorie restriction and structured exercise on reductions in visceral and abdominal subcutaneous adipose tissue: A systematic review. Eur. J. Clin. Nutr..

[26] Rostami H (2017). Habitual dietary intake of fatty acids are associated with leptin gene expression in subcutaneous and visceral adipose tissue of patients without diabetes. Prostaglandins Leukot. Essent. Fatty Acids.

[27] Yuzbashian E (2019). Determinants of vitamin D receptor gene expression in visceral and subcutaneous adipose tissue in non-obese, obese, and morbidly obese subjects. J. Steroid Biochem. Mol. Biol..

[28] Committee, I. R. Guidelines for data processing and analysis of the International Physical Activity Questionnaire (IPAQ)-short and long forms. (2005). http://www.ipaq.ki.se/scoring.pdf.

[29] Vasheghani-Farahani A (2011). The Persian, last 7-day, long form of the international physical activity questionnaire: Translation and validation study. Asian J. Sports Med..

[30] Mahmoodi B, Shemshaki A, Zarkesh M, Hedayati M, Mirmiran P (2019). Habitual physical activity is associated with relative apelin gene expression in adipose tissues among non-diabetic adults. Int. J. Pept. Res. Ther..

[31] Livak KJ, Schmittgen TD (2001). Analysis of relative gene expression data using real-time quantitative PCR and the 2−ΔΔCT method. Methods.

[32] Sylow L, Kleinert M, Richter EA, Jensen TE (2017). Exercise-stimulated glucose uptake—Regulation and implications for glycaemic control. Nat. Rev. Endocrinol..

[33] Chadt A, Al-Hasani H (2020). Glucose transporters in adipose tissue, liver, and skeletal muscle in metabolic health and disease. Pflügers Arch. Eur. J. Physiol..

[34] Ferrannini E (2018). Adipose tissue and skeletal muscle insulin-mediated glucose uptake in insulin resistance: Role of blood flow and diabetes. Am. J. Clin. Nutr..

[35] Flores-Opazo M, Raajendiran A, Watt MJ, Hargreaves M (2019). Exercise serum increases GLUT4 in human adipocytes. Exp. Physiol..

[36] Fruman DA (2017). The PI3K pathway in human disease. Cell.

[37] Marinou K (2014). Structural and functional properties of deep abdominal subcutaneous adipose tissue explain its association with insulin resistance and cardiovascular risk in men. Diabetes Care.

[38] Mittal B (2019). Subcutaneous adipose tissue & visceral adipose tissue. Indian J. Med. Res..

[39] Ibrahim MM (2010). Subcutaneous and visceral adipose tissue: Structural and functional differences. Obes. Rev..

[40] Virtanen KA (2002). Glucose uptake and perfusion in subcutaneous and visceral adipose tissue during insulin stimulation in nonobese and obese humans. J. Clin. Endocrinol. Metab..

[41] Hannukainen JC (2010). Higher free fatty acid uptake in visceral than in abdominal subcutaneous fat tissue in men. Obesity.

[42] Wedell-Neergaard A-S (2019). Exercise-induced changes in visceral adipose tissue mass are regulated by IL-6 signaling: A randomized controlled trial. Cell Metab..

[43] Bertholdt L, Gudiksen A, Ringholm S, Pilegaard H (2020). Impact of skeletal muscle IL-6 on subcutaneous and visceral adipose tissue metabolism immediately after high-and moderate-intensity exercises. Pflügers Arch. Eur. J. Physiol..

[44] Macpherson RE, Huber JS, Frendo-Cumbo S, Simpson JA, Wright DC (2015). Adipose tissue insulin action and IL-6 signaling after exercise in obese mice. Med. Sci. Sports Exerc..

[45] Li L (2021). Interleukin-6 mediated exercise-induced alleviation of adiposity and hepatic steatosis in mice. BMJ Open Diabetes Res. Care.

[46] Trinh B (2021). Blocking endogenous IL-6 impairs mobilization of free fatty acids during rest and exercise in lean and obese men. Cell Rep. Med..

[47] Meng Q (2020). IRS1/PI3K/AKT pathway signal involved in the regulation of glycolipid metabolic abnormalities by Mulberry (*Morus alba* L.) leaf extracts in 3T3-L1 adipocytes. Chin. Med..

[48] Esfahani M, Movahedian A, Baranchi M, Goodarzi MT (2015). Adiponectin: An adipokine with protective features against metabolic syndrome. Iran. J. Basic Med. Sci..

[49] Polito R (2020). The important role of adiponectin and orexin-A, two key proteins improving healthy status: Focus on physical activity. Front. Physiol..

[50] Pereira RI, Draznin B (2005). Inhibition of the phosphatidylinositol 3′-kinase signaling pathway leads to decreased insulin-stimulated adiponectin secretion from 3T3-L1 adipocytes. Metabolism.

[51] Su X (2021). PI3K/Akt pathway expression in children with different obesity degrees and its relationship with glucolipid metabolism and insulin resistance. Am. J. Transl. Res..

[52] Hosseini Khorami SA (2020). Genetic determinants of obesity heterogeneity in type II diabetes. Nutr. Metab..

[53] Świderska E (2018). Role of PI3K/AKT pathway in insulin-mediated glucose uptake. Blood Glucose Levels.

